# Inhibitory Activity of Marine Sponge-Derived Natural Products against Parasitic Protozoa

**DOI:** 10.3390/md8010047

**Published:** 2010-01-15

**Authors:** Ilkay Orhan, Bilge Şener, Marcel Kaiser, Reto Brun, Deniz Tasdemir

**Affiliations:** 1 Department of Pharmacognosy, Faculty of Pharmacy, Gazi University, TR-06330 Ankara, Turkey; E-Mails: iorhan@gazi.edu.tr (I.O.); bilgesen@gazi.edu.tr (B. Ş.); 2 Department of Medical Parasitology and Infection Biology, Swiss Tropical Institute, CH-4002 Basel, Switzerland; E-Mails: marcel.kaiser@unibas.ch (M.K.); reto.brun@unibas.ch (R.B.); 3 Department of Pharmaceutical and Biological Chemistry, School of Pharmacy, University of London, London WC1N 1AX, UK

**Keywords:** marine sponge, Spongia, Ircinia, antiprotozoal activity, Trypanosoma, Leishmania, Plasmodium

## Abstract

In this study, thirteen sponge-derived terpenoids, including five linear furanoterpenes: furospinulosin-1 (**1**), furospinulosin-2 (**2**), furospongin-1 (**3**), furospongin-4 (**4**), and demethylfurospongin-4 (**5**); four linear meroterpenes: 2-(hexaprenylmethyl)-2-methylchromenol (**6**), 4-hydroxy-3-octaprenylbenzoic acid (**7**), 4-hydroxy-3-tetraprenyl-phenylacetic acid (**8**), and heptaprenyl-*p*-quinol (**9**); a linear triterpene, squalene (**10**); two spongian-type diterpenes dorisenone D (**11**) and 11β-acetoxyspongi-12-en-16-one (**12**); a scalarane-type sesterterpene; 12-*epi*-deoxoscalarin (**13**), as well as an indole alkaloid, tryptophol (**14**) were screened for their *in vitro* activity against four parasitic protozoa; *Trypanosoma brucei rhodesiense*, *Trypanosoma cruzi*, *Leishmania donovani* and *Plasmodium falciparum*. Cytotoxic potential of the compounds on mammalian cells was also assessed. All compounds were active against *T. brucei rhodesiense*, with compound **8** being the most potent (IC_50_ 0.60 μg/mL), whereas **9** and **12** were the most active compounds against *T. cruzi*, with IC_50_ values around 4 μg/mL. Compound **12** showed the strongest leishmanicidal activity (IC_50_ 0.75 μg/mL), which was comparable to that of miltefosine (IC_50_ 0.20 μg/mL). The best antiplasmodial effect was exerted by compound **11** (IC_50_ 0.43 μg/mL), followed by compounds **7**, **10**, and **12** with IC_50_ values around 1 μg/mL. Compounds **9**, **11** and **12** exhibited, besides their antiprotozoal activity, also some cytotoxicity, whereas all other compounds had low or no cytotoxicity towards the mammalian cell line. This is the first report of antiprotozoal activity of marine metabolites **1**–**14**, and points out the potential of marine sponges in discovery of new antiprotozoal lead compounds.

## 1. Introduction

It is estimated that over one billion people suffer from one or more tropical diseases. Among them, protozoal diseases caused by single-celled parasites are of particular importance. With the highest rates of complications and mortality, severe malaria, caused by the most fatal species *Plasmodium falciparum*, is the most dangerous protozoal disease. There were 247 million cases of malaria in 2006, causing nearly one million deaths, mostly among African children [1]. Key interventions to control malaria include prompt and effective treatment with artemisinin-based combination therapies (ACT), use of insecticide-treated nets by people at risk and indoor residual spraying with insecticide to control the vector, female *Anopheles* mosquitoes [2]. The resistance developed against safe insecticides and recent report of resistance against ACT in Southeast Asia [3], underlines the urgent need for novel chemotherapeutics against malaria. Apart from malaria, African trypanosomiasis, leishmaniasis and Chagas disease represent three most important diseases caused by parasitic protozoa. It is estimated that these three diseases are responsible for more than 110,000 deaths every year [4]. African trypanosomiasis (sleeping sickness) is caused by the parasitic flagellates; *Trypanosoma brucei gambiense* and *T. brucei rhodesiense*, which invade the central nervous system, leading to behavioral changes, coma, and ultimately, if left untreated, death [5]. Chagas’ disease, or South American trypanosomiasis, is caused by *T. cruzi* and occurs primarily in young children. The intermediate host is a blood-sucking insect (triatome), which bites the victim and contaminates the wound with infected feces. Visceral leishmaniasis is another group of parasitic disease caused by *Leishmania donovani*, which is transmitted to humans by sand flies [6]. In the absence of a long-term protecting vaccine, the control of these parasitic infections is based on a few chemotherapeutic agents, most of which were discovered in the 1950s and whose mechanisms of action are not completely known. Furthermore, they are often associated with severe adverse effects and variable efficacy according to the phase of the disease. Also the emerging resistance against these drugs makes the discovery and development of new, safe and effective antiprotozoal agents an urgent need.

**Figure 1:**
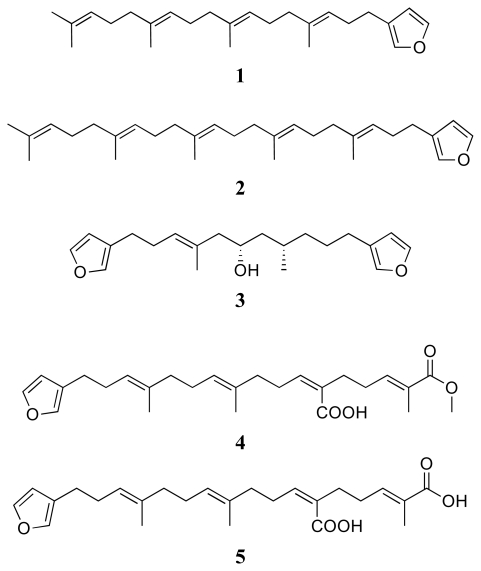

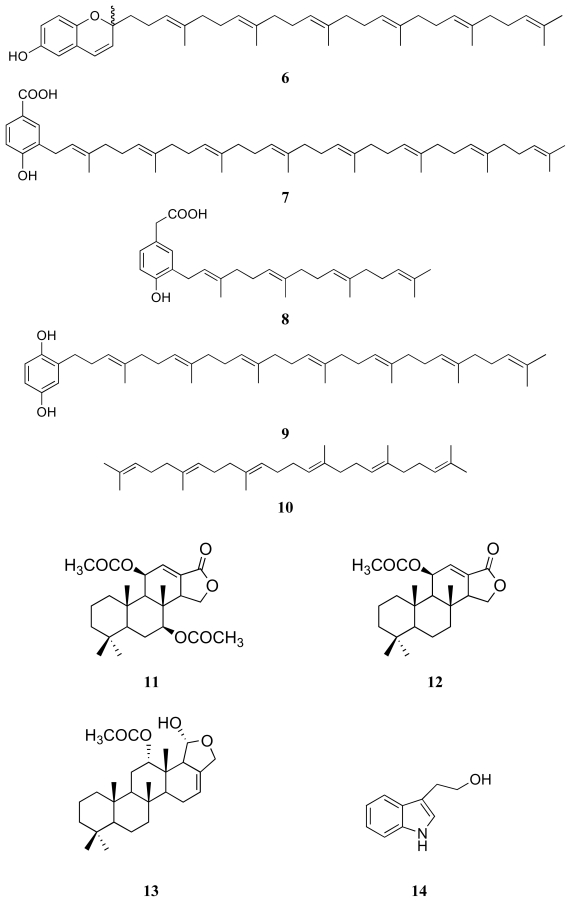
Chemical structures of compounds **1**–**14.**

The intensive research since 1970s has proved that marine organisms are magnificent sources of bioactive secondary metabolites. In some excellent reviews, a number of the compounds originating from marine organisms have been reported to possess *in vitro* and *in vivo* antiprotozoal activity [7–10]. In the continuation of our project concerning the evaluation of the anti-infective activity of Turkish marine organisms and their secondary metabolites [11], herein we report the broad-spectrum antiprotozoal activity and cytotoxicity of fourteen compounds, which we previously isolated from the marine sponges *Spongia* sp. and *Ircinia* sp. collected from the Turkish coastline of the Aegean Sea. The majority of the compounds are terpenoids (Figure 1) and consist of five linear furanoterpenes: furospinulosin-1 (**1**), furospinulosin-2 (**2**), furospongin-1 (**3**), furospongin-4 (**4**), and demethylfurospongin-4 (**5**); four linear meroterpenes: 2-(hexaprenylmethyl)-2-methylchromenol (**6**), 4-hydroxy-3-octaprenylbenzoic acid (**7**), 4-hydroxy-3-tetraprenylphenylacetic acid (**8**), and heptaprenyl-*p*-quinol (**9**); a linear triterpene, squalene (**10**); two spongian-type diterpenes: dorisenone D (**11**) and 11β-acetoxyspongi-12-en-16-one (**12**); a scalarane sesterterpene: 12-*epi*-deoxoscalarin (**13**), plus the indole alkaloid tryptophol (**14**). The metabolites were evaluated by medium-throughput screening methods for their *in vitro* activity against the mammalian stage of four parasitic protozoa; *Trypanosoma brucei rhodesiense* (bloodstream forms), *Trypanosoma cruzi* (intracellular amastigotes in L6 rat skeletal myoblasts), *Leishmania donovani* (axenic amastigotes), and *Plasmodium falciparum* (blood stage forms of K1 strain resistant to chloroquine and pyrimethamine). In order to assess the selectivity indices of the compounds, they were also tested towards a mammalian cell line (rat skeletal myoblasts: L6 cells). Melarsoprol, benznidazole, miltefosine, chloroquine and podophyllotoxin were used as reference drugs. This is the first study reporting the inhibitory effects of compounds **1**–**14** against parasitic protozoa.

## 2. Results and Discussion

Marine sponge-originated compounds **1**–**14** showed very promising *in vitro* antiprotozoal activities. As shown in Table 1, all metabolites displayed some *in vitro* antitrypanosomal activity against *T. b. rhodesiense* with great variations in the IC_50_ values, which ranged from 0.60 μg/mL to 55.25 μg/mL. The best inhibition against this protozoan parasite was displayed by 4-hydroxy-3-tetraprenylphenylacetic acid (**8**) (IC_50_ 0.60 μg/mL), followed by dorisenone D (**11**, IC_50_ 2.47 μg/mL), heptaprenyl-*p*-quinol (**9**, IC_50_ 3.54 μg/mL), 11β-acetoxyspongi-12-en-16-one (**12**, IC_50_ 4.14 μg/mL), demethylfurospongin-4 (**5**) (IC_50_ 4.90 μg/mL) and tryptophol (**14**, IC_50_ 5.89 μg/mL). On the other hand, the best-inhibiting compounds towards *T. cruzi* were found to be heptaprenyl-*p*-quinol (**9**, IC_50_ 4.08 μg/mL), and 11β-acetoxyspongi-12-en-16-one (**12**, IC_50_ 4.51 μg/mL). Only three of the remaining compounds, **3**, **13** and **14**, showed anti-*Trypanosoma cruzi* activity with higher IC_50_ values. Trypanocidal activity profile of furospongin-1 (**3**) and 12-*epi*-deoxoscalarin (**13**) against both *Trypanosoma* species was almost identical. However, tryptophol (**14**), the only non-terpenoid marine natural product tested, was 8-fold less activite against *T. cruzi* (IC_50_ 49.37 μg/mL) than against *T. b. rhodesiense*. The majority of the metabolites also showed growth inhibitory activity against *L. donovani*. Among all active substances, 11β-acetoxyspongi-12-en-16-one (**12**) exerted a highly remarkable inhibition against *L. donovani* with an IC_50_ value of 0.75 μg/mL, which was comparable to that of the reference compound, miltefosine (IC_50_ 0.20 μg/mL). Furospongin-1 (**3**) and 4-hydroxy-3-octaprenylbenzoic acid (**7**) also displayed notable antileishmanial activity with IC_50_ values of 4.80 and 5.60 μg/mL, respectively, whereas the remaining active compounds had moderate IC_50_ values ranging from 9.60 μg/mL to 18.9 μg/mL. Except for 2-(hexaprenylmethyl)-2-methylchromenol (**6**) and heptaprenyl-*p*-quinol (**9**), all metabolites screened in this study displayed some antiplasmodial activity against the multi-drug resistant strain of *P. falciparum* (Table 1). The best inhibition was exhibited by dorisenone D (**11**, IC_50_ 0.43 μg/mL). Also 11β-acetoxyspongi-12-en-16-one (**12**), squalene (**10**), and 4-hydroxy-3-octaprenylbenzoic acid (**7**) showed significant activity with IC_50_ values of 1.09, 1.16 and 1.57 μg/mL, respectively. The IC_50_ values of the remaining eight active metabolites were in a narrower range and varied between 3.30 μg/mL and 14.02 μg/mL. From the evaluation of the metabolites against mammalian L6 cells, some interesting results became apparent. As shown in Table 1, the IC_50_ values of compounds **9**, **11** and **12** against mammalian cells were very similar to their IC_50_ values against the parasitic protozoa. Although there are variations in the antiprotozoal activity against different protozoa of interest, the toxicity against mammalian cells might still indicate a lack of selective toxicity, i.e. general toxicity, for these compounds. However, the most potent trypanocidal compound, 4-hydroxy-3-tetraprenylphenylacetic acid (**8**) was devoid of any cytotoxicity even at the highest test concentrations (90 μg/mL). The remaining compounds had either low or no cytotoxic potential (IC_50_ > 90 μg/mL).

Protozoal diseases continue to be one of the major health problems worldwide and since protozoa develop resistance quickly to currently available drug series, the discovery of novel antiprotozoal agents has become a pressing need. A number of chemically diverse phytochemicals (e.g., alkaloids, terpenes, quinones, flavonoids, aurones, xanthones, and cyclic tetrapeptides) with antiprotozoal activity have been reported in the literature [12,13]. Several types of marine natural products, such as manzamine alkaloids, plakortin type acetogenins and cyano/isocyano/formamide containing sesqui- and diterpenes have been studied *in vitro* and/or *in vivo* for antiprotozoal (mostly antimalarial) activity [8–10], but the real potential of marine organisms has remained largely uninvestigated. Hence, this study aimed at focusing on the *in vitro* antiprotozoal activity of a variety of sponge terpenoids, including di-, sester-, and triterpenes, as well as various length of polyterpenes and meroterpenes, which were never tested against any of the protozoa of interest. This study is a part of a large screening campaign we have recently initiated on marine extracts/metabolites and has already yielded some promising compounds as shown in this study.

Linear furanoterpenes **1**–**5** studied here showed a moderate to good, but generally broad-spectrum growth inhibitory activity against almost all parasites and were devoid of significant cytotoxicity. Anti-infective activity of several linear furanoterpenes is known [14], but to our knowledge, their antiparasitic activity has not been explored in detail. Compounds **1** and **2** are structurally very similar, except that **2** contains an additional isoprene unit. The length of isoprene chain appears to have an impact on antiparasitic potentials of linear furanoterpenes, as **2** shows better *T. b. rhodesiense* and *P. falciparum activity*, but fails to inhibit *L. donovani* cultures. Again the presence of an additional isoprene unit, as in the case of **2**, leads to some cytotoxicity. There were also some differences in the antiprotozoal potential of furanoterpenes **4** and **5**, which differ from each other by the presence of a terminal methyl group. Interestingly, furospongin-1 (**3**), a C-21 furanoterpene with two furane rings and a hydroxyl function is active against all parasite strains and bears some cytotoxicity.

Meroterpene-derivatives (**6**–**9**) had also significant levels of biological activity on the parasitic protozoa tested. In several previous studies, some meroterpene-type compounds were reported to have antimalarial activity in μM range [15–17], which is consistent with the results obtained in the present study. 4-Hydroxy-3-tetraprenylphenylacetic acid (**8**) has emerged as the most potent compound against African trypanosomes without any cytotoxicity. Interestingly, a related compound, heptaprenyl-*p*-quinol (**9**), arrested the growth of African and American trypanosomes equipotently with moderate IC_50_ values. However, **9** also inhibited the growth of mammalian cells, indicating a narrow therapeutic window. Some sponge-derived quinol derivatives have been reported to exert cytotoxic activity [18,19], the mechanism of which has been associated with the oxidation of quinol to quinone. The meroterpenes **7**–**9** have structural similarities in their linearity, however it is obvious that the presence of a shorter isoprenyl chain and a phenylacetic acid as a terminal unit, as found in compound **8**, is most favored for *T. b. rhodesiense* activity and highest selectivity. The linear triterpene squalene (**10**) appeared to have good antiplasmodial activity, plus some moderate activity against *T. brucei rhodesiense*. To our knowledge, squalene has never been reported to exhibit antiplasmodial or antitrypanosomal activity up to date.

Spongian diterpenoids are natural products isolated mainly from sponges and nudibranchs, which feed on these sponges [20]. It has been speculated that the oxygenated positions of spongian-diterpenoids act as a complexing moiety for cations and such complexation might play a role in their biological activity [21]. The spongian diterpene dorisenone D (**11**) was found to be the best antiplasmodial agent with some efficacy against *T. brucei rhodesiense*. For an antimalarial hit, the WHO Special Program for Research and Training in Tropical Diseases (TDR) defines an activity criterion to have an IC_50_ value < 0.2 μg/mL [22]. In our study, dorisenone D (**11**) emerged to be the closest one to this criterion. Towards the malaria parasite, dorisenone D (**11**) has a selectivity index of >9 (calculated by: IC_50_ against L6 cells/IC_50_ against parasite). If the cytotoxicity of **11** can be lowered by medicinal chemistry approach, dorisenone D could become a promising antiplasmodial lead compound. The second spongian diterpene 11β-acetoxyspongi-12-en-16-one (**12**) exhibited the highest antileishmanial potential, however, its selectivity index for *L. donovani* was very low (4.4). It was also toxic towards all other parasites, pointing out rather a general toxicity. Compound **12** is the 7-deacetyl derivative of **11**, however there are very significant differences in their antiparasitic activity. As shown in Table 1, it seems that the absence of an acetoxy function at C-7 position, as in **12**, is favored for particularly for *T. cruzi* and *L. donovani* activity. Notably, both compounds show almost identical cytotoxicity towards mammalian cells, which might suggest that other shared structural characteristics are responsible for cytotoxicity. *Plasmodium falciparum* was the most susceptible parasite for 12-*epi*-deoxoscalarin (**13**), the only scalaran-terpene investigated herein.

Many indole-derivatives from natural sources have been reported with antiprotozoal activity [23–26]. Tryptophol (3-indole ethanol) (**14**), a plant auxin derivative, which was also isolated from a marine sponge for the first time by our group [27], showed a broad-spectrum activity versus *T. brucei rhodesiense*, *L. donovani* and *P. falciparum* without eliciting high cytotoxicity.

## 3. Experimental Section

### 3.1. Isolation of compounds **1–14**

Compounds **1**–**14** were isolated from the marine sponges *Spongia* sp. and *Ircinia* sp. collected from the Turkish coastline of the Aegean Sea using various chromatographic techniques as described previously and characterized by means of spectroscopic techniques [27,28]. The purity of compounds (>95%) was confirmed by ^1^H- and ^13^C-NMR.

### 3.2. Activity against Plasmodium falciparum

*In vitro* activity against erythrocytic stages of *P. falciparum* was determined by a modified [^3^H]-hypoxanthine incorporation assay [29], using the chloroquine- and pyrimethamine-resistant K1 strain and the standard drug artemisinin. Briefly, parasite cultures incubated in RPMI 1640 medium with 5% Albumax (without hypoxanthine) were exposed to serial drug dilutions in microtiter plates. After 48 h of incubation at 37°C in a reduced oxygen atmosphere, 0.5 μCi ^3^H-hypoxanthine was added to each well. Cultures were incubated for a further 24 h before they were harvested onto glass-fiber filters and washed with distilled water. The radioactivity was counted using a Betaplate^™^ liquid scintillation counter (Wallac, Zurich). The results were recorded as counts per minute (CPM) per well at each drug concentration and expressed as percentage of the untreated controls. IC_50_ values were calculated from the sigmoidal inhibition curves using Microsoft Excel. Chloroquine was the reference drug used.

### 3.3. Activity against Trypanosoma brucei rhodesiense

*Trypanosoma brucei rhodesiense*, STIB 900 strain, and the standard drug, melarsoprol, were used for the assay. This stock was isolated in 1982 from a human patient in Tanzania and after several mouse passages cloned and adapted to axenic culture conditions. Minimum Essential Medium (50 μL) supplemented with 25 mM HEPES, 1g/L additional glucose, 1% MEM non-essential amino acids (100x), 0.2 mM 2-mercaptoethanol, 1 mM Na-pyruvate and 15% heat inactivated horse serum was added to each well of a 96-well microtiter plate [30,31]. Serial drug dilutions of seven 3-fold dilution steps covering a range from 90 to 0.123 μg/mL were prepared. Then 10^4^ bloodstream forms of *T. b. rhodesiense* STIB 900 in 50 μL was added to each well and the plate incubated at 37 °C under a 5% CO_2_ atmosphere for 72 h. 10 μL Alamar Blue (resazurin, 12.5 mg in 100 mL double-distilled water) was then added to each well and incubation continued for a further 2–4 h [32]. Then, the plates were read with a Spectramax Gemini XS microplate fluorometer (Molecular Devices Cooperation, Sunnyvale, CA, USA) using an excitation wavelength of 536 nm and an emission wavelength of 588 nm. Data were analyzed using the microplate reader software Softmax Pro (Molecular Devices Cooperation, Sunnyvale, CA, USA).

### 3.4. Activity against Trypanosoma cruzi

Rat skeletal myoblasts (L6 cells) were seeded in 96-well microtitre plates at 2000 cells/well in 100 μL RPMI 1640 medium with 10% FBS and 2 mM l-glutamine. After 24 h the medium was removed and replaced by 100 μL per well containing 5000 trypomastigote forms of *T. cruzi* Tulahuen strain C2C4 containing the β-galactosidase (Lac Z) gene [33]. After 48 h, the medium was removed from the wells and replaced by 100 μL fresh medium with or without a serial drug dilution of seven 3-fold dilution steps covering a range from 90 to 0.123 μg/mL. After 96 h of incubation, the plates were inspected under an inverted microscope to assure growth of the controls and sterility. Then, the substrate CPRG/Nonidet (50 μL) was added to all wells. A color reaction developed within 2–6 h and could be read photometrically at 540 nm. Data were transferred into the graphic programme Softmax Pro (Molecular Devices), which calculated IC_50_ values. Benznidazole was the reference drug used.

### 3.5. Activity against Leishmania donovani

Amastigotes of *L. donovani* strain MHOM/ET/67/L82 were grown in axenic culture at 37 °C in SM medium at pH 5.4 supplemented with 10% heat-inactivated fetal bovine serum under an atmosphere of 5% CO_2_ in air. One hundred microlitres of culture medium with 10^5^ amastigotes from axenic culture with or without a serial drug dilution were seeded in 96-well microtitre plates. Serial drug dilutions covering a range from 90 to 0.123 μg/mL were prepared. After 72 h of incubation the plates were inspected under an inverted microscope to assure growth of the controls and sterile conditions. 10 μL of Alamar Blue (12.5 mg resazurin dissolved in 100 mL distilled water) [34] were then added to each well and the plates incubated for another 2 h. Then, the plates were read with a Spectramax Gemini XS microplate fluorometer using an excitation wavelength of 536 nm and an emission wavelength of 588 nm. Data were analyzed using the software Softmax Pro (Molecular Devices Cooperation, Sunnyvale, CA, USA). Decrease of fluorescence (=inhibition) was expressed as percentage of the fluorescence of control cultures and plotted against the drug concentrations. From the sigmoidal inhibition curves the IC_50_ values were calculated. Miltefosine was used as reference drug.

### 3.6. Cytotoxicity against L6 cells

Assays were performed in 96-well microtiter plates, each well containing 100 μL of RPMI 1640 medium supplemented with 1% l-glutamine (200 mM) and 10% fetal bovine serum, and 4 × 10^4^ L6 cells (a primary cell line derived from rat skeletal myoblasts). Serial drug dilutions of seven 3-fold dilution steps covering a range from 90 to 0.123 μg/mL were prepared. After 72 h of incubation, the plates were inspected under an inverted microscope to assure growth of the controls and sterile conditions. Ten μL of Alamar Blue solution was then added to each well and the plates incubated for another 2 h. Then the plates were read with a Spectramax Gemini XS microplate fluorometer using an excitation wavelength of 536 nm and an emission wavelength of 588 nm. Data were analysed using the microplate reader software Softmax Pro. Podophyllotoxin was the reference drug used.

## 4. Conclusions

This is the first report of antiprotozal activity assessments of fourteen sponge metabolites. The results demonstrate that some of the compounds, such as 4-hydroxy-3-tetraprenylphenylacetic acid (**8**), can be considered as an interesting hit against parasitic diseases. Besides **8**, dorisenone D (**11**) and 11β-acetoxyspongi-12-en-16-one (**12**) may also be seen as hits, after their selectivity is increased by rational drug design process. Further investigations may include the assessment of underlying mechanisms of action of these compounds and evaluation of their *in vivo* efficacy in animal models, which could not be performed in the current study due to low available amounts of the compounds.

## Figures and Tables

**Table 1:** *In vitro* antiprotozoal and cytotoxic activities of sponge-derived compounds **1**–**14**. The IC_50_ values are in μg/mL and represent the average of at least two independent assays performed in duplicates.

Compound	*Trypanosoma b. rhodesiense*	*Trypanosoma cruzi*	*Leishmania donovani*	*Plasmodium falciparum*	Cytotoxicity

**1**	27.45	>90	14.2	11.18	>90
**2**	16.16	>90	>90	3.51	34.0
**3**	13.94	17.49	4.80	14.02	27.45
**4**	17.94	>90	>90	7.51	>90
**5**	4.90	>90	10.2	13.36	>90
**6**	41.43	>90	15.9	>20	>90
**7**	13.11	>90	5.60	1.57	86.48
**8**	**0.60**	>90	>90	3.30	>90
**9**	3.54	**4.08**	18.9	>20	**2.62**
**10**	15.03	>90	>90	1.16	34.89
**11**	2.47	>90	>90	**0.43**	**3.93**
**12**	4.14	**4.51**	**0.75**	1.09	**3.32**
**13**	55.25	40.43	>90	7.48	60.33
**14**	5.89	49.37	9.60	5.08	63.46

Standards	0.003^a^	0.359^b^	0.20^c^	0.056^d^	0.004^e^

Reference compounds

a: melarsoprol,

b: benznidazole,

c: miltefosine,

d: chloroquine,

e: podophyllotoxin.
